# Reduced *Plasmodium* Parasite Burden Associates with CD38^+^ CD4^+^ T Cells Displaying Cytolytic Potential and Impaired IFN-γ Production

**DOI:** 10.1371/journal.ppat.1005839

**Published:** 2016-09-23

**Authors:** Julie G. Burel, Simon H. Apte, Penny L. Groves, Kerenaftali Klein, James S. McCarthy, Denise L. Doolan

**Affiliations:** 1 Molecular Vaccinology Laboratory, QIMR Berghofer Medical Research Institute, Brisbane, Australia; 2 The University of Queensland, School of Medicine, Brisbane, Australia; 3 Statistics Unit, QIMR Berghofer Medical Research Institute, Brisbane, Australia; 4 Clinical Tropical Medicine Laboratory, QIMR Berghofer Medical Research Institute, Brisbane, Australia; 5 Centre for Biosecurity and Tropical Infectious Diseases, Australian Institute of Tropical Health and Medicine, James Cook University, Cairns, Australia; McGill University, CANADA

## Abstract

Using a unique resource of samples from a controlled human malaria infection (CHMI) study, we identified a novel population of CD4^+^ T cells whose frequency in the peripheral blood was inversely correlated with parasite burden following *P*. *falciparum* infection. These CD4^+^ T cells expressed the multifunctional ectoenzyme CD38 and had unique features that distinguished them from other CD4^+^ T cells. Specifically, their phenotype was associated with proliferation, activation and cytotoxic potential as well as significantly impaired production of IFN-γ and other cytokines and reduced basal levels of activated STAT1. A CD38^+^ CD4^+^ T cell population with similar features was identified in healthy uninfected individuals, at lower frequency. CD38^+^ CD4^+^ T cells could be generated *in vitro* from CD38^-^ CD4^+^ T cells after antigenic or mitogenic stimulation. This is the first report of a population of CD38^+^ CD4^+^ T cells with a cytotoxic phenotype and markedly impaired IFN-γ capacity in humans. The expansion of this CD38^+^ CD4^+^ T population following infection and its significant association with reduced blood-stage parasite burden is consistent with an important functional role for these cells in protective immunity to malaria in humans. Their ubiquitous presence in humans suggests that they may have a broad role in host-pathogen defense.

**Trial Registration:**

ClinicalTrials.gov clinical trial numbers ACTRN12612000814875, ACTRN12613000565741 and ACTRN12613001040752

## Author Summary

Malaria is one of the three most deadly infectious disease worldwide, together with tuberculosis and HIV. The exact mechanisms underlying effective immunity to malaria remain largely unknown and there is no reliable immune correlate of protection. Here, we take advantage of a unique experimental human infection model to define the immune response to primary exposure of blood-stage malaria parasites in naïve healthy volunteers at the molecular level. We report that *Plasmodium* parasite levels were inversely correlated to the expansion of a specific subset of CD4^+^ T cells expressing the activation molecule CD38 and a very unusual phenotype. Although the expansion of CD38^+^ CD4^+^ T cells has been described in several viral and bacterial infections, we show for the first time that these cells are associated with a ‘naive-like’ effector phenotype, higher cytolytic potential and a strongly impaired ability to produce IFN-γ and other cytokines. Importantly, this subset of CD38^+^ CD4^+^ T cells could be also identified in all healthy volunteers prior to infection, suggesting that these core characteristics of circulating CD38^+^ CD4^+^ T cells are independent of active infection and may play an important role in the immune control of other pathogens.

## Introduction

Malaria is associated with complex multi-factorial immune responses, due in part to the multi-stage life cycle of the *Plasmodium* spp. parasite which is targeted by multiple arms of the immune system, and the existence of elaborate host-pathogen interactions and evasion mechanisms [1]. The effector cells and immune mediators contributing to protection against the sporozoite, liver, and blood-stages of malaria have been the subject of intense investigation over many years [2–4], but the specific molecular mechanisms and critical effector cells that mediate control of parasite burden remain largely unknown [1,3,5].

CD4^+^ T cells have been implicated in the control of blood-stage parasitemia in numerous animal models [6]; and in humans an association with parasite control has been demonstrated in studies utilizing CHMI [7,8]. An important effector function of CD4^+^ T cells is the production of various pro- and anti-inflammatory cytokines including IFN-γ, IL-2, IL-4, IL-10, IL-17 and TNF [9]. In blood-stage malaria, IFN-γ has been implicated as the key cytokine driving effective immune responses [10], and circumstantial evidence associates CD4^+^ T cells producing IFN-γ with protection against *P*. *falciparum* blood-stage infection in humans [7,8]. CD4^+^ T cells expressing the degranulation marker CD107a have been identified in circulating PBMCs from subjects protected by immunization with *P*. *falciparum* sporozoites under the cover of chloroquine chemoprophylaxis following *in vitro* stimulation with *P*. *falciparum*–infected red blood cells [11]. Cytolytic CD4^+^ T cells have also been described as potent immune effectors in protection against many viral infections, including CMV, Epstein-Barr Virus, Influenza and HIV [12–14].

In humans, recently activated T cells are typically identified *ex vivo* via the upregulation of cell surface glycoproteins such as CD69 or CD38 [15]. CD69 is one of the earliest molecules detected on the T cell surface following TCR engagement, and CD69 mediated signaling results in a range of cellular responses including proliferation [16]. CD38 is expressed on the surface of immature but not mature hematopoietic cells, and is also re-expressed by many immune cells (including CD4^+^ T cells) after activation [17,18]. Circulating T cells expressing CD38 during infection have been associated with a recently activated effector phenotype [19,20]; and antigen-specific CD4^+^ T cells have been shown to express high levels of CD38 and produce IFN-γ in the acute phase of several viral infections including EBV [21], HIV [22] and *Influenza* [23]. CD38 is a multifunctional molecule that acts as an ectoenzyme to catalyze the conversion of NAD^+^ to the second-messenger cyclic-ADB-ribose (cADPR) and is considered the major regulator of intracellular and extracellular NAD^+^ levels [24]. The purpose of CD38 signaling in T cell immunity is not fully elucidated, although there is strong evidence that the products of CD38 enzymatic activity (principally cADPR) enhance intracellular calcium mobilization which is required for a multitude of cell functions including lymphocyte proliferation [25] and lymphocyte migration [26,27]. Signaling through CD38 has also been reported to promote the activation and cytotoxic function of human NK cells [28,29].

Experimental infection models provide an excellent opportunity to investigate the mechanisms underlying the induction of immunity following primary exposure of the human immune system to target pathogens. Here, we utilized a model of controlled human malaria infection (CHMI) of naive healthy volunteers [30–32] to explore the mechanisms underlying the induction of T-cell-associated immunity in blood-stage malaria. We show that the levels of parasite burden following experimental *P*. *falciparum* blood-stage infection inversely correlated with the expansion of a specific subset of CD4^+^ T cells expressing CD38 and characterized by high cytotoxic potential but impaired cytokine production. These cells were also present at lower frequency in healthy uninfected individuals, and could be generated *in vitro* from CD38^-^ CD4^+^ T cells after antigenic or mitogenic stimulation.

## Results

### Control of parasite burden following first-exposure to *P*. *falciparum* correlates with the expansion of CD38^+^ CD4^+^ T cells

**Fig 1:**
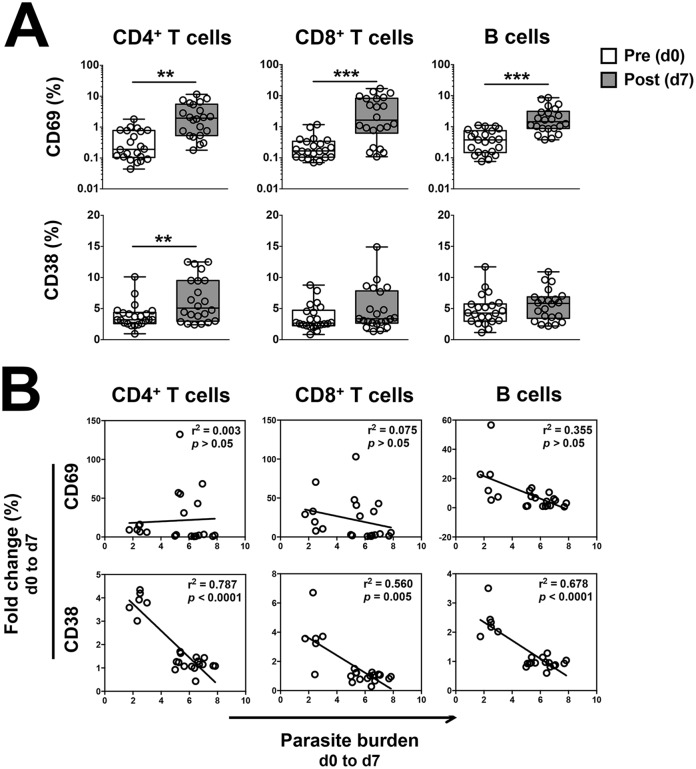
Changes in the frequency of CD69^+^ and CD38^+^ T cells and B cells circulating during *P*. *falciparum* blood-stage infection and their association with parasite burden. Peripheral blood was collected prior to and seven days post-infection of malaria-naïve individuals with ~ 1800 pRBCs and the frequency of CD69^+^ or CD38^+^ T cells and B cells determined by flow cytometry. Parasite burden was calculated as the Area Under the Curve from blood parasite levels measured by qPCR from day 0 to day 7 post-infection. **(A)** Frequency of CD69^+^ or CD38^+^ T cells and B cells circulating pre- and post-infection. **(B)** Univariate regression analysis of the changes in frequency of CD69^+^ or CD38^+^ T cells and B cells circulating during infection and parasite burden. Statistical differences between cell frequencies pre- and post-infection were determined using the non-parametric Wilcoxon test; box and whisker plots indicate median, interquartile range and min-max. Univariate regression analysis was performed with R, and *p* values adjusted for multiple comparisons using the Bonferroni method. Graphs show combined data from 22 volunteers from five independent cohorts. **, *p* < 0.01; ***, *p* < 0.001.

To determine whether significant changes could be detected in the phenotype of circulating lymphocytes following infection, the frequencies of T cells and B cells expressing the activation markers CD69 or CD38 were measured prior to and seven days post-infection with *P*. *falciparum*. In all volunteers, infection led to a significant increase in the frequency and absolute numbers of CD69^+^ T cells and B cells (Fig 1A and S1 Fig). There was a higher frequency of CD38^+^ CD4^+^ T cells but not CD38^+^ CD8^+^ T cells or B cells (Fig 1A), and absolute numbers of CD38^+^ CD4^+^ T cells and B cells also increased in the peripheral blood during infection (S1 Fig). In the same volunteers, measurement of the level of parasitemia by qPCR over the first seven days of infection revealed a variation in parasite burden between individuals. Thus, we compared the changes in frequency of CD69^+^ and CD38^+^ T cells and B cells during infection with parasite burden, to determine if a certain cell type or phenotype was associated with parasite control. Univariate regression analysis revealed no significant association between the changes in the frequency of CD69^+^ T cells or B cells and the parasite burden during infection (Fig 1B, *top panel*). However, an inverse correlation between the frequency of CD38^+^ T cells and B cells and parasite burden during infection was apparent (Fig 1B, *bottom panel*). Multivariate regression analysis with forward selection revealed that the fold change in the frequency of CD38^+^ in CD4^+^ T cells was the only variable significantly negatively associated with parasite burden (*p* = 7.5E-05). Thus, in malaria-naive volunteers, reduced parasite burden during primary blood-stage *P*. *falciparum* infection was associated with the expansion of a specific subset of CD4^+^ T cells expressing the activation marker CD38.

### CD38^+^ CD4^+^ T cells in *P*. *falciparum* infected individuals have a ‘naïve-like’ effector phenotype associated with proliferation, activation and cytolytic potential

**Fig 2:**
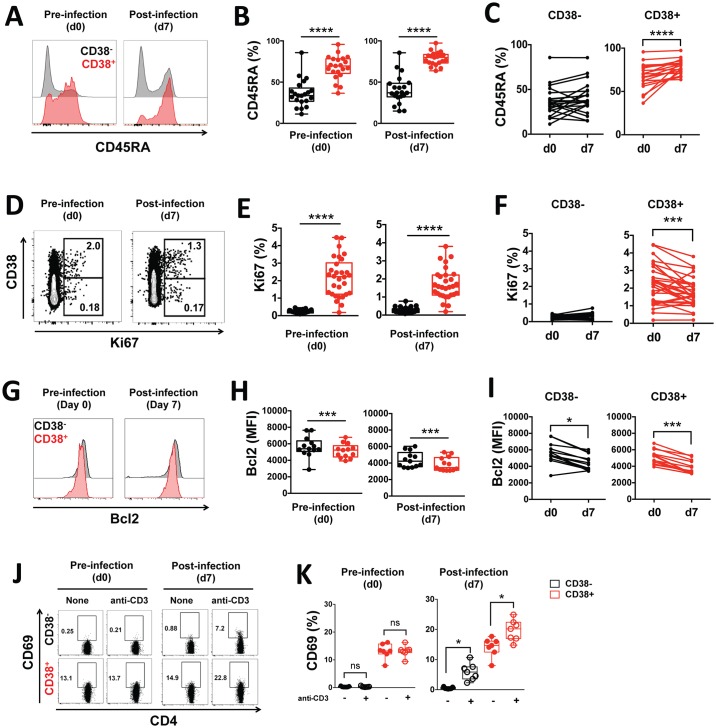
CD38^+^ CD4^+^ T cells have a ‘naïve-like’ effector phenotype associated with proliferation and recent activation. **(A)** Representative staining of one volunteer and **(B-C)** the frequencies of CD45RA^+^ cells in CD38^-^ (black) or CD38^+^ (red) CD4^+^ T cells determined by flow cytometry in volunteers prior to and seven days post infection. **(D)** Representative staining of one volunteer and **(E-F)** the frequencies of Ki67^+^ cells in CD38^-^ (black) or CD38^+^ (red) CD4^+^ T cells determined by flow cytometry in volunteers prior to and seven days post infection. **(G)** Representative staining of one volunteer and **(H-I)** the average expression of Bcl2 in CD38^-^ (black) or CD38^+^ (red) CD4^+^ T cells determined by flow cytometry in volunteers prior to and seven days post infection. **(J)** Representative staining of one volunteer and **(K)** the frequencies of CD69^+^ cells measured by flow cytometry in CD38^-^ (black) or CD38^+^ (red) CD4^+^ T cells freshly isolated from volunteers prior to and seven days post infection after brief stimulation with plate-bound anti-human CD3 antibody. Graphs show combined data from (A-F) 22 volunteers from five independent cohorts, (G-I) 13 volunteers from two independent cohorts, (J-K) seven volunteers from one cohort. Statistical differences between CD38^-^ and CD38^+^ CD4^+^ T cells or between pre- and post-infection were determined using the non-parametric Wilcoxon test; box and whisker plots indicate median, interquartile range and min-max; *, *p* < 0.05; **, *p* < 0.01; ***, *p* < 0.001; ****, *p* < 0.0001 ns, *p* > 0.05.

In order to further delineate the differentiation state of CD38^+^ CD4^+^ T cells, we measured their surface expression of the naïve marker CD45RA. Similarly to previous reports [33], we found that a greater proportion of CD38^+^ CD4^+^ T cells expressed CD45RA in comparison to CD38^-^ CD4^+^ T cells both prior to, and at peak infection (Fig 2A and 2B). Furthermore, there was an increased in the frequency of CD45RA^+^ cells at 7 days post infection compared to prior to infection in CD38^+^ but not in CD38^-^ CD4^+^ T cells (Fig 2C).

Effector T cell populations expanding in response to infection or immunization have been shown to express high levels of CD38 and the proliferation marker Ki67 but low levels of the anti-apoptotic molecule Bcl2 [19]. Accordingly, we measured the expression of Ki67 and Bcl2 by flow cytometry within CD38^+^ and CD38^-^ CD4^+^ T cells circulating in the healthy volunteers prior to infection, as well as at peak of infection seven days after inoculation. The expression of Ki67 was significantly increased in CD38^+^ CD4^+^ T cells compared to CD38^-^ CD4^+^ T cells prior to and during infection (*p* < 0.0001, Fig 2D and 2E). Additionally, the frequency of Ki67^+^ cells decreased at 7 days post infection compared to prior to infection in CD38^+^ but not in CD38^-^ CD4^+^ T cells (Fig 2F). Conversely, the expression of the anti-apoptotic marker Bcl2 was significantly reduced in CD38^+^ CD4^+^ T cells compared to CD38^-^ CD4^+^ T cells both prior to and at peak infection (*p* = 0.002, Fig 2G and 2H). In both cell subsets, there was an overall decrease in Bcl2 expression at 7 days post infection compared to prior to infection, but to a greater extent for CD38^+^ CD4^+^ T cells (Fig 2I). Thus, CD38^+^ CD4^+^ T cells were associated with a ‘naïve-like’ effector T cell phenotype (CD45RA^+^Ki67^+^Bcl2^low^), and the proportion of cells expressing each marker was significantly affected upon infection within this cell subset.

As CD38 upregulation has been observed following TCR-signaling of CD4^+^ T cells [21–23], we reasoned that CD38^+^ CD4^+^ T cells might have increased activation following TCR *in vitro* stimulation compared to CD38^-^ CD4^+^ T cells. We assessed the level of activation of the FACS-purified CD38^+^ and CD38^-^ CD4^+^ T cells via TCR stimulation by measuring their capacity to upregulate CD69 expression after short *in vitro* stimulation with anti-CD3. Prior to infection, there was no significant increase in CD69 expression following TCR stimulation for both CD38^+^ and CD38^-^ CD4^+^ T cells (Fig 2J and 2K), although the basal expression of CD69 was higher on CD38^+^ CD4^+^ T cells. Post-infection, while both cell types significantly upregulated CD69 expression following TCR stimulation (Fig 2J and 2K), fold change was significantly higher for CD38^-^ CD4^+^ T cells compared to CD38^+^ CD4^+^ T cells (average fold change of 13.7 and 1.4 for CD38^-^ CD4^+^ T cells and CD38^+^ CD4^+^ T cells, respectively, *p* = 0.016). However, the higher basal expression of CD69 for CD38^+^ CD4^+^ T cells was maintained. These results suggest that *P*. *falciparum* blood-stage infection of humans led to an increased level of activation following TCR stimulation in both CD38^+^ and CD38^-^ CD4^+^ cell subsets, albeit to a greater extent for CD38^-^ CD4^+^ T cells. Furthermore, CD38^+^ CD4^+^ T cells constitutively express higher levels of CD69 compared to CD38^-^ CD4^+^ T cells.

**Fig 3:**
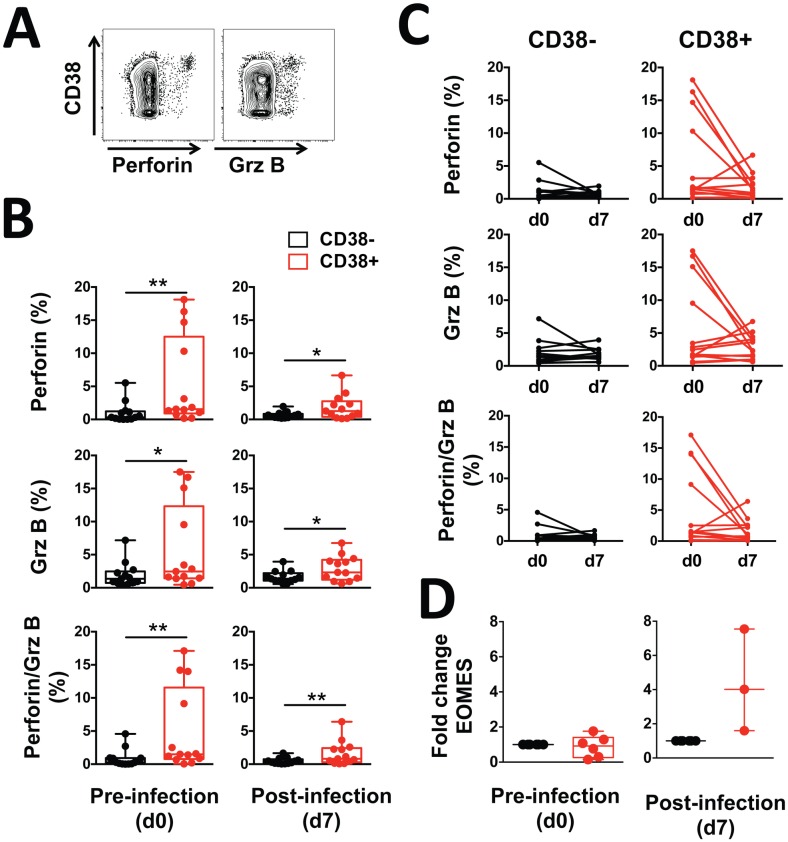
CD38^+^ CD4^+^ T cells have a phenotype associated with cytotoxic potential. (A) Representative staining in one volunteer and (B-C) frequencies of CD38^-^ (black) or CD38^+^ (red) CD4^+^ T cells expressing perforin^+^, granzyme B^+^ (GrzB) or perforin^+^/granzyme B^+^ determined by flow cytometry in volunteers prior to and seven days post infection. **(D)** Eomes gene expression measured by RT-qPCR in CD38^-^ (black) or CD38^+^ (red) CD4^+^ T cells isolated from volunteers prior to and seven days post infection. Graphs show combined data from (A-C) 13 volunteers from two independent cohorts, (D) seven (pre) and three (post) volunteers from one cohort. Statistical differences between CD38^-^ and CD38^+^ CD4^+^ T cells or between pre- and post-infection were determined using the non-parametric Wilcoxon test; box and whisker plots indicate median, interquartile range and min-max; *, *p* < 0.05; **, *p* < 0.01; ***, *p* < 0.001; ****, *p* < 0.0001.

A recent report associated CD4^+^ T cells expressing the degranulation marker CD107a with protection against malaria in sporozoite-immunized volunteers [11], and CD38 has been implicated in the cytotoxic activity of NK cells [28,29]. Thus, we examined the cytolytic potential of CD38^+^ CD4^+^ T cells by measuring their intracellular expression of perforin and granzyme B by flow cytometry both prior to and at peak infection. The frequency of cells expressing perforin or granzyme B was significantly greater in CD38^+^ CD4^+^ T cells than in CD38^-^ CD4^+^ T cells prior to and post infection (*p* = 0.001 and *p* = 0.011 prior to infection, and *p* = 0.012 and *p* = 0.017 at peak infection for perforin and granzyme B, respectively, Fig 3A and 3B). Additionally, the CD38^+^ CD4^+^ T cells contained a higher proportion of cells co-expressing perforin and granzyme B (*p* = 0.001 and *p* = 0.006 prior to and at peak infection, respectively, (Fig 3B). The proportion of cells expressing granzyme B and/or perforin within either CD38^+^ or CD38^-^ CD4^+^ T cell subsets did not change markedly during the course of infection (Fig 3C). Eomesodermin (Eomes), a key transcription factor associated with the cytolytic activity of CD4^+^ T cells [34,35] was expressed at similar levels by CD38^+^ and CD38^-^ CD4^+^ T cells prior to infection. However, a trend for an increase in Eomes expression in CD38^+^ CD4^+^ T cells following infection was observed (Fig 3D). Taken together, these results suggest that the CD38^+^ CD4^+^ T cell subset that expands during *P*. *falciparum* infection defines an effector population with cytolytic potential.

### CD38^+^ CD4^+^ T cells are a subset of conventional TCR-αβ CD4^+^ T cells

**Fig 4:**
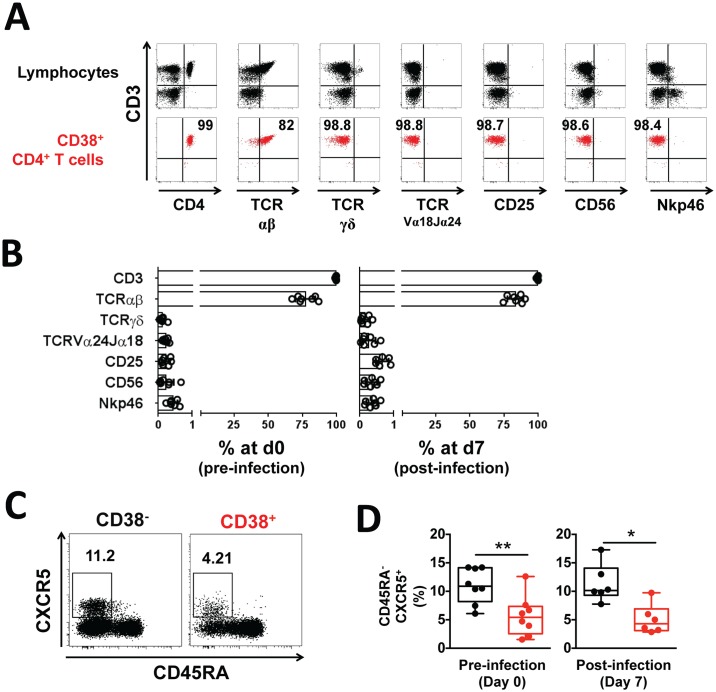
CD38^+^ CD4^+^ T cells are a subset of conventional TCR-αβ CD4^+^ T cells. Peripheral blood was collected prior to and seven days post-infection and the lineage phenotype of CD38^+^ CD4^+^ T cells determined by flow cytometry. **(A)** Representative staining of CD3, CD4, TCR-αβ, TCR-γδ, TCR-Vα24Jα18, CD25, CD56 and Nkp46 within CD38^+^ CD4^+^ T cells (red) or total lymphocytes (grey) from one volunteer. **(B)** Mean frequency of CD3, TCR-αβ, TCR-γδ, TCR-Vα24Jα18, CD25, CD56 and Nkp46 positive cells within CD38^+^ CD4^+^ T cells circulating prior to and seven days post-infection. Graphs show combined data from seven volunteers from one cohort; error bars indicate SD. (C) Concatenated plot representing the frequency of CXCR5^+^CD45RA^-^ cells within CD38^+^ CD4^+^ T cells or CD38^-^ CD4^+^ T cells from 6 volunteers at seven days post infection. (D) Mean frequency of CXCR5^+^CD45RA^-^ cells within CD38^+^ CD4^+^ T cells (red) or CD38^-^ CD4^+^ T cells (black) prior to and seven days post infection. Graphs show combined data from eight (pre) and six (post) volunteers from one cohort. Statistical differences between CD38^-^ and CD38^+^ CD4^+^ T cells were determined using the non-parametric Wilcoxon test; box and whisker plots indicate median, interquartile range and min-max; *, *p* < 0.05; **, *p* < 0.01.

We next confirmed that the circulating CD38^+^ CD4^+^ T cells belong to a conventional CD4^+^ T cell subset and not to another immune cell type known to have a strong cytotoxic activity, such as NK cells or NKT cells. The CD38^+^ CD4^+^ T cells were all positive for CD3 expression (99.5±0.5%, marker for T cell lineage) and mainly positive for TCR-αβ expression (83.4±5.8%, marker for αβ T cells) but were negative for TCR-γδ expression (0.16±0.1%, marker for γδ T cells [36]), TCR-Vα24Jα18 expression (0.25±0.2%, marker for NK T cells [37]), CD25 expression (0.93±0.8%, marker for regulatory T cells [38]), as well as CD56 and Nkp46 expression (0.35±0.2% and 0.36±0.2%, respectively, markers for NK cells [39]) (Fig 4A and 4B). Thus, circulating CD38^+^ CD4^+^ T cells represent a subset of conventional TCR-αβ CD4^+^ T cells.

T follicular helper (Tfh) cells are conventional CD4^+^ T cells responsible for the generation of long-lived and high affinity antibody responses [40]. Activation and expansion of a specific subset of Tfh cells was observed in the peripheral blood of children experiencing acute malaria infection [41]. Both prior to and at peak infection, the frequency of CD45RA^-^CXCR5^+^ Tfh cells was significantly higher amongst CD38^-^ CD4^+^ T cells compared to CD38^+^ CD4^+^ T cells (Fig 4C and 4D), and Tfh cells represented a small fraction of the total CD38^+^ CD4^+^ T cell population (average of 5.6% and 5% of total CD38^+^ CD4^+^ T cells prior to, and at peak infection, respectively, Fig 4C and 4D). Thus, circulating CD38^+^ CD4^+^ T cells that expand during infection do not represent a specific subset of Tfh cells.

### CD38^+^ CD4^+^ T cells are poor producers of IFN-γ

**Fig 5:**
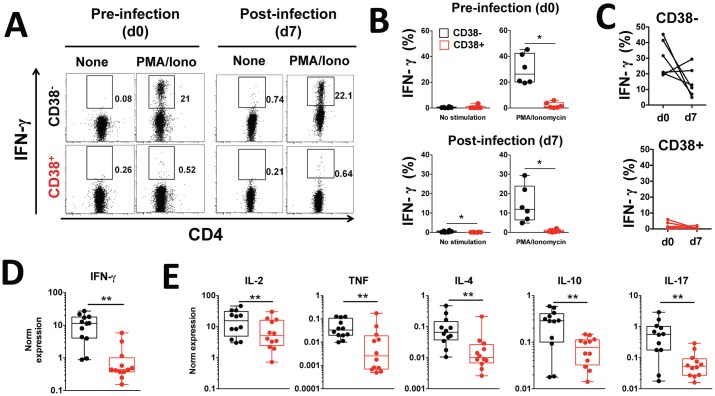
CD38^+^ CD4^+^ T cells are poor producers of IFN-γ following *in vitro* stimulation. **(A-C)** Peripheral blood was collected prior to and seven days post-infection and CD38^-^ (black) and CD38^+^ (red) CD4^+^ T cells isolated using cell sorting. Intracellular IFN-γ production after brief mitogenic stimulation with PMA and Ionomycin was measured at the protein level using flow cytometry. **(D-E)** Peripheral blood was collected from healthy uninfected volunteers and **(D)** IFN-γ and **(E)** IL-2, TNF, IL-4, IL-10 and IL-17 gene expression after brief mitogenic stimulation with PMA and Ionomycin was measured by RT-qPCR. Gene expression was normalized to reference gene RPL13A. Graphs show combined data from (A-C) four to seven volunteers from one cohort and (D-E) 12 healthy volunteers; statistical differences between CD38^+^ and CD38^-^ CD4^+^ T cells and between prior to and seven days post infection were determined using the non-parametric Wilcoxon test; box and whisker plots indicate median, interquartile range and min-max; *, *p* < 0.05, **, *p* < 0.01; ***, *p* < 0.001; ****, *p* < 0.0001.

IFN-γ is considered the key effector cytokine in malaria [10], and cytolytic CD4^+^ T cells have been shown to produce high levels of IFN-γ upon *in vitro* stimulation [42]. To compare the ability of CD38^+^ and CD38^-^ CD4^+^ T cells to produce IFN-γ, we assessed the frequency of IFN-γ positive cells induced by mitogenic stimulation in CD38^+^ and CD38^-^ CD4^+^ T cell populations collected prior to and at peak infection seven days later. Unexpectedly, IFN-γ production was significantly impaired in the CD38^+^ CD4^+^ T cell subset both prior to and during infection (*p* = 0.031 and *p* = 0.031) (Fig 5A and 5B). For both cell subsets, infection did not trigger changes in their capacity to produce IFN-γ in response to stimulation (Fig 5C). The impairment of IFN-γ production in CD38^+^ CD4^+^ T cells is apparently controlled at the level of gene expression as IFN-γ mRNA was significantly reduced in mitogen-stimulated CD38^+^ CD4^+^ T cells taken from healthy uninfected volunteers when compared to their CD38^-^ CD4^+^ T cells (Fig 5D), and was also apparent for several other cytokines including IL-2, TNF, IL-4, IL-10 and IL-17 (Fig 5E).

Since the CD38^+^ CD4^+^ T cell population displayed a phenotype associated with recent activation combined with a marked inability to produce cytokines, we next confirmed that the CD38^+^ CD4^+^ T cells were not undergoing apoptosis through an activation-induced cell death mechanism [43]. CD38^+^ CD4^+^ T cells stained negatively for the apoptotic marker Annexin-V and the viability stain propidium iodide (Pi) prior to infection (98.4±0.5% Annexin-V^-^, 99.8±0.5% Pi^-^) and during infection (98.8±0.8% Annexin-V^-^, 99.8±0.2% Pi^-^). Thus, the CD38^+^ CD4^+^ T cell population contains viable cells that are not undergoing apoptosis.

### Reduced levels of activated pSTAT1 in CD38^+^ CD4^+^ T cells

**Fig 6:**
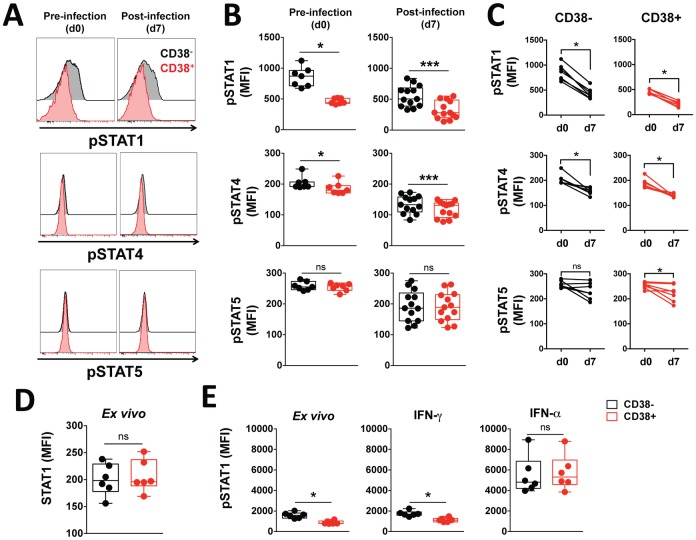
CD38^+^ CD4^+^ T cells have a different STAT phosphorylation profile compared to CD38^-^ CD4^+^ T cells. **(A)** Representative staining of one volunteer and **(B-C)** the expression levels of pSTAT1, pSTAT4 and pSTAT5 in CD38^-^ (black) or CD38^+^ (red) CD4^+^ T cells measured *ex vivo* by phospho-epitope specific flow cytometry in volunteers prior to and seven days post infection. **(D)** The expression level of total STAT1 protein in CD38^-^ (black) or CD38^+^ (red) CD4^+^ T cells from healthy uninfected volunteers measured by flow cytometry. **(E)** The expression level of pSTAT1 in CD38^-^ (black) or CD38^+^ (red) CD4^+^ T cells from healthy uninfected volunteers measured by phospho-epitope specific flow cytometry *ex vivo* or after 15 mins stimulation with 100 ng/mL human recombinant IFN-γ or IFN-α measured by phospho-epitope specific flow cytometry. Differences between CD38^-^ and CD38^+^ CD4^+^ T cells and between prior to and seven days post infection were determined with the non-parametric Wilcoxon test; box and whisker plots indicate median, interquartile range and min-max. Graphs show combined data from (A-C) seven volunteers from one cohort (pre) and 13 volunteers from two separate cohorts (post), (D-E) six healthy controls. *, *p* < 0.05; ***, *p* < 0.001 ns, *p* > 0.05.

To determine whether the impaired ability of CD38^+^ CD4^+^ T cells to produce IFN-γ and other cytokines was due to a specific defect in cytokine-associated signaling pathways, we investigated the level of phosphorylated STATs within CD38^+^ CD4^+^ T cells using Phosphoflow [44]. STAT1 is mainly phosphorylated through stimulation with type I or type II IFNs [45], and a critical role for STAT4 activation by type I IFNs in the IFN-γ response has been reported [46]. Consistent with this, CD38^+^ CD4^+^ T cells displayed significantly reduced levels of both pSTAT1 and pSTAT4 compared to CD38^-^ CD4^+^ T cells (*p* = 0.016 and *p* = 0.031 prior to infection, and *p* = 0.0002 and *p* = 0.0002 post-infection for pSTAT1 and pSTAT4, respectively; Fig 6A and 6B). Unexpectedly, since STAT5 mediates IL-2 signaling in T cells [47] and we found that IL-2 production by CD38^+^ CD4^+^ T cells was significantly reduced compared to CD38^-^ CD4^+^ T cells (Fig 5E), the levels of pSTAT5 were similar between CD38^+^ and CD38^-^ CD4^+^ T cells (Fig 6A and 6B), suggesting that both cells subsets retain similar capacity to respond to IL-2. Upon infection, the levels of pSTAT1 and pSTAT4 within both CD38^+^ and CD38^-^ CD4^+^ T cells decreased (Fig 6C), while the expression of pSTAT5 was significantly decreased in the CD38^+^ CD4^+^ T cell subset only (Fig 6C).

Differences in the basal levels of pSTAT1 between the two CD4^+^ T cell subsets were not due to differences in total STAT1 protein as there was no apparent difference in the levels of intracellular STAT1 between CD38^-^ and CD38^+^ CD4^+^ T cells (Fig 6D).

In order to determine whether the observed differences in basal levels of pSTAT1 were due to variation in cytokine responsiveness, we assessed the phosphorylation levels of STAT1 in CD38^+^ and CD38^-^ CD4^+^ T cells isolated from healthy uninfected volunteers following *in vitro* exposure to IFN-γ or IFN-α. In both cell subsets, a 15 min exposure to IFN-γ induced a small but insignificant increase in phosphorylation of STAT1 (Fig 6E). However, IFN-α exposure induced significant phosphorylation of STAT1 (both *p* = 0.0313, with *non-parametric Wilcoxon test*) and brought the levels of pSTAT1 to similar levels in the CD38- and CD38^+^ subsets (Fig 6E), indicating a similar responsiveness to IFN-α and ability to phosphorylate STAT1 and excluding a specific defect in the ability of CD38^+^ T cells to phosphorylate STAT1.

### CD38^-^ CD4^+^ T cells have the ability to differentiate into CD38^+^ CD4^+^ T cells upon *in vitro* stimulation

**Fig 7:**
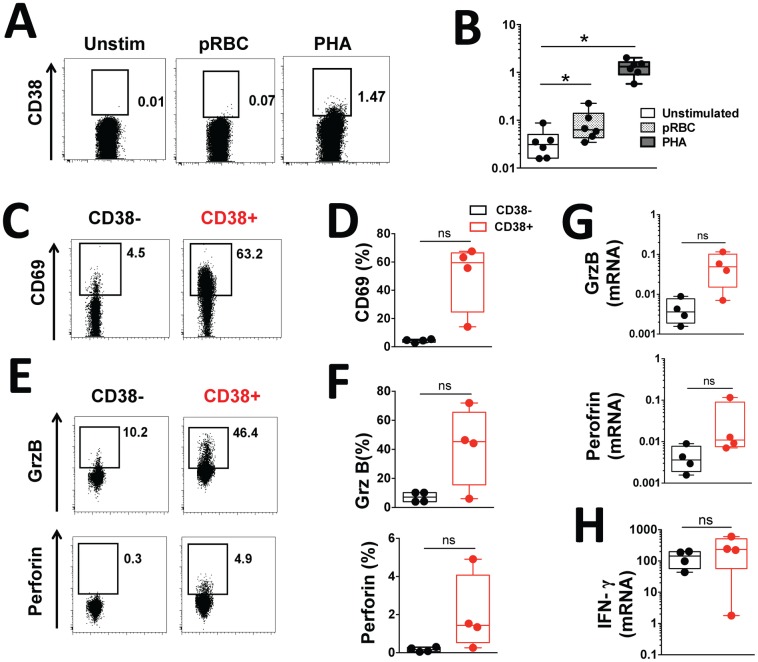
CD38^-^ CD4^+^ T cells can differentiate into CD38^+^ CD4^+^ T cells upon *in vitro* stimulation. **(A)** Representative staining of one volunteer and **(B)** the frequency of CD38^+^ cells measured by flow cytometry in CD38^-^ CD4^+^ T cells isolated from healthy volunteers and *in vitro* stimulated with *P*. *falciparum* parasitized red blood cells extract (pRBC) at 10^6^ pRBC/mL or PHA at 5 μg/mL for seven days. **(C-H)** CD38^-^ CD4^+^ T cells were isolated from healthy volunteers, *in vitro* stimulated for seven days with 5 μg/mL PHA and sorted into CD38^-^ and CD38^+^ CD4^+^ T cells for flow cytometry or gene expression analysis. **(C)** Representative staining of one volunteer and **(D)** the frequencies of CD69^+^ cells measured by flow cytometry in CD38^-^ (black) or CD38^+^ (red) CD4^+^ T cells. **(E)** Representative staining of one volunteer and **(F)** the frequencies of Perforin^+^ and Granzyme B^+^ cells determined by flow cytometry in CD38^-^ (black) or CD38^+^ (red) CD4^+^ T cells. **(G)** Gene expression of Perforin and Granzyme B measured by RT-qPCR in CD38^-^ (black) or CD38^+^ (red) CD4^+^ T cells. **(H)** IFN-γ gene expression measured by RT-qPCR after PMA and Ionomycin stimulation of CD38^-^ (black) or CD38^+^ (red) CD4^+^ T cells. Gene expression was normalized to reference gene RPL13A. Graphs show combined data from (A-B) six and (C-H) four healthy volunteers. Differences between culture conditions were determined using the non-parametric Wilcoxon test; box and whisker plots indicate median, interquartile range and min-max; *, *p*<0.05 ns, p > 0.05.

Finally, we investigated the cell subset precursor of the CD38^+^ CD4^+^ T cells that expanded *in vivo* upon infection. Since there was no change in absolute numbers of CD4^+^ T cells in the peripheral blood of infected volunteers during infection (S2 Fig), we hypothesized that the increase in the frequency and absolute numbers of CD38^+^ CD4^+^ T cells circulating during infection might result from the differentiation of naive CD4^+^ T cells into activated CD38^+^ CD4^+^ T cells, rather than from the proliferation of pre-existing CD38^+^ CD4^+^ T cells. To test this, isolated CD38^-^ CD4^+^ T cells from healthy uninfected volunteers were stimulated *in vitro* for 7 days, and then assayed for expression of CD38 by flow cytometry. Consistent with our hypothesis, stimulation with both parasite antigen and mitogen resulted in a significant upregulation of CD38 (Fig 7A and 7B). Similar to the CD38^+^ CD4^+^ T cells circulating *in vivo* in the *P*. *falciparum* experimental infection model, this *in vitro* generated CD38^+^ CD4^+^ T cell population showed a phenotype associated with recent activation (Fig 7C and 7D) and cytotoxic potential at both protein (Fig 7E and 7F) and mRNA levels (Fig 7G). Unlike endogenous CD38^+^ CD4^+^ T cells, these *in vitro* generated CD38^+^ CD4^+^ T cells had similar capacity to produce IFN-γ (Fig 7H) as well as other cytokines (S3 Fig) upon *in vitro* stimulation.

## Discussion

Herein, we identify a novel population of CD38^+^ CD4^+^ T cells displaying cytolytic potential and a phenotype associated with proliferation and activation. Increased frequency of CD38^+^ CD4^+^ T cells in the peripheral blood of human volunteers inversely correlated with parasite burden in an experimental blood-stage malaria infection model. Importantly, these CD38^+^ CD4^+^ T cells had a significantly reduced ability to produce IFN-γ and several other cytokines, and contained reduced basal levels of phosphorylated STAT1, compared to CD38^-^ CD4^+^ T cells. This CD38^+^ CD4^+^ T cell population was also present at low frequency in all healthy uninfected individuals we tested. CD38^+^ CD4^+^ T cells with cytolytic potential could be generated *in vitro* from CD38^-^ CD4^+^ T cells following exposure to antigen or mitogen but did not display impaired cytokine expression.

The CD38^+^ CD4^+^ T cells we described in this study have features of both naïve and effector T cells, but are distinguished by their strongly impaired cytokine function. These results are in concordance with the previous study of Plebanski *et al*. describing a specific subset of circulating CD4^+^ T cells expressing CD38 and CD45RA but with reduced proliferation and Th1 cytokine production upon TCR stimulation [33]. In our study, the CD38^+^ CD4^+^ T cells circulating during infection displayed a phenotype associated with recent activation and cytotoxic potential, suggesting that this cell population may be a subset of cytolytic CD4^+^ T cells. We are aware of one early report of cytotoxic CD4^+^ T cell generation in a human volunteer following experimental immunization irradiated *P*. *falciparum* sporozoites [48] and protection from sporozoite infection conferred by adoptive transfer of cytolytic CD4^+^ T cell clones in a rodent model [49]. Both of those examples utilized the liver-stage of the parasite where the host cells include MHC-II expressing Kupffer cells and hepatocytes and there is solid evidence that cytolytic CD4^+^ T cells can induce death in target cells through MHC-II dependent mechanisms, thereby contributing to protection against pathogens with a tropism for MHC-II expressing cells [9,12]. However, since red blood cells lack MHC-II molecules, so the mechanism by which CD38^+^ CD4^+^ T cells could exert their cytotoxic function in blood-stage malaria remains to be elucidated. One possibility is that the cells might be operating through a MHC-independent mechanism, as observed in NK cells [50]. Alternatively, the cytolytic CD38^+^ CD4^+^ T cells might be responsible for MHC-II dependent killing of recently activated antigen presenting cells (APCs) in order to prevent the development of exacerbated pro-inflammatory responses. Indeed, it has been demonstrated that CD4^+^ T cells can mediate both cytotoxic and suppressor functions *in vitro* [51] and that cytolytic CD4^+^ T cells can directly induce apoptosis of activated APCs [52]. Thus, we speculate that the function of our novel population of CD38^+^ CD4^+^ T cells during infection might be to control and restrain the immune response, rather than to promote it.

Unexpectedly, we found that these CD38^+^ CD4^+^ T cells are very poor producers of IFN-γ in response to mitogenic stimulation, compared to CD38^-^ CD4^+^ T cells. This result was surprising because IFN-γ has been implicated as the main effector cytokine produced by CD4^+^ T cells and contributing to protective immunity to blood-stage malaria [6,10]. Furthermore, CD4^+^ T cells displaying a cytotoxic function are thought to co-express IFN-γ [42,53,54]. To our knowledge, our study provides the first evidence of a differential IFN-γ production capability and cytotoxic phenotype between CD38^+^ and CD38^-^ circulating CD4^+^ T cells in humans, and of a disassociation between cytotoxicity and IFN-γ production.

Additionally, this CD38^+^ CD4^+^ T cell population had a limited ability to produce other pro and anti-inflammatory cytokines including TNF, IL-2, IL-4, IL-10 and IL-17. This reduced capacity of CD38^+^ CD4^+^ T cells to produce cytokines does not appear to be due to exhaustion or a form of activation induced cell death since these cells could be activated following TCR stimulation and were negative for Annexin-V. Thus, CD38^+^ CD4^+^ T cells display a phenotype of functionally active T cells with reduced capacity to produce cytokines, notably IFN-γ. These observations, together with previous studies on regulatory T cells that identified a positive association between CD38 expression on CD4^+^ T cells and immune suppressive activity [55,56], further support our hypothesis that CD38^+^ CD4^+^ T cells might have a repressive regulatory function rather than a direct cytotoxic activity against malaria parasites or infected cells.

The CD38^+^ CD4^+^ T cells displayed low basal levels of pSTAT1, which is a key transcription factor associated with IFN-γ signaling [45,57], suggesting that their reduced IFN-γ production might be associated with a defect in STAT1-signalling pathway. We found that the marked reduction in basal levels of phosphorylated STAT1 in CD38^+^ CD4^+^ T cells is not due to a defect in responsiveness to IFN-α or to a capability to phosphorylate STAT1, nor could the differences be accounted for in basal levels of total STAT1 protein. Thus, constitutively lower levels of activated STAT1 within this cell population might explain the reduced expression of activated STAT1 measured *ex vivo* within CD38^+^ CD4^+^ T cells. Alternatively, the difference in pSTAT1 basal levels between CD38^+^ and CD38^-^ CD4^+^ T cells might be due to differences in cytokine responsiveness other than type I or type II IFNs. Indeed, STAT1 can also be phosphorylated through IL-6, IL-10 and IL-21 [58]. A more comprehensive cytokine signaling profiling and STAT responsiveness of CD38^+^ CD4^+^ T cells in comparison to other CD4^+^ T cell subsets could address this question.

CD38^+^ CD4^+^ T cells retained their cytotoxic function and impaired ability to produce cytokines throughout infection. Although infection led to significant changes in the phenotype of both CD38^+^ and CD38^-^ CD4^+^ T cell subsets during infection, there was a marked increase in CD45RA expression and decrease in Ki67 expression within CD38^+^ but not CD38^-^ CD4^+^ T cells. Hence, we speculate that, upon infection, ‘naïve-like’ CD38^+^ CD4^+^ T cells proliferate and differentiate into parasite-specific effector memory CD4^+^ T cells, which are then recruited out of the peripheral blood and into lymphoid organs such as the spleen where they specifically exert their cytotoxic function. Consistent with this is our observation that there were no changes in the cytotoxic phenotype of circulating CD38^+^ CD4^+^ T cells were observed in the volunteers following infection.

Both the absolute numbers of CD38^+^ in CD4^+^ T cells and relative frequency of CD38^+^ versus CD38^-^ CD4^+^ T cell were increased upon infection. Thus, the accumulation of CD38^+^ CD4^+^ T cells in the peripheral blood upon infection is not due to the recruitment of CD38^-^ CD4^+^ T cell subsets into other body compartments such as lymphoid organs. Increases in frequency of CD38^+^ CD4^+^ T cells can originate from the activation of circulating CD38^-^ CD4^+^ T cells already present in the peripheral blood prior to infection, as suggested in Fig 7. Alternatively, newly generated CD38^+^ CD4^+^ T cells might be originating from other lymphoid organs such as the spleen and found in the peripheral blood as a ‘spillover’ effect. We found that the proportion of CD45RA^+^ within CD38^+^ but not CD38^-^ CD4^+^ T cells is significantly increased upon infection (Fig 2C) and CD38 has been shown to be preferentially expressed by CD45RA^+^ CD4^+^ T cells and might play a role for lymphocyte homing on those cells [33]. Thus CD38^+^ and CD38^-^ CD4^+^ T cells might have differential homing properties, so distinct abilities to migrate between lymphoid organs.

In contrast to CD38^+^ CD4^+^ T cells that expanded in experimentally infected volunteers, the *in vitro* generated CD38^+^ CD4^+^ T cells were fully capable of producing IFN-γ and other cytokines in response to stimulation. Thus, the reduced ability of CD38^+^ CD4^+^ T cells to produce IFN-γ is a unique feature of CD38^+^ cells that arise *in vivo*. Cytokine production by T cells is highly modulated during T cell differentiation, notably through epigenetic modifications [59–61]. Thus, it is possible that *in vivo* the upregulation of CD38 by CD4^+^ T cells is accompanied by epigenetic changes that inhibit the transcription of genes coding for cytokines. Thus, we speculate that the analysis of epigenetic modifications to cytokine promoters in circulating CD38^+^ CD4^+^ T cells in the context of natural or experimental infection will inform the molecular mechanisms controlling cytokine production within this cell population.

Although an increase in frequency of circulating CD38^+^ CD4^+^ T cells has been observed following immunization or infection in a number of host-pathogen systems [20,23,62], the role of those cells has not been elucidated, and impaired cytokine capacity has not been reported. All volunteers in our CHMI study had a pre-existing proportion of CD4^+^ T cells expressing CD38 prior to infection, as do all healthy volunteers we have since examined. All CD38^+^ CD4^+^ T cells isolated from healthy volunteers either prior to infection, or during experimental malaria infection, displayed similar features clearly distinguishing them from CD38^-^ CD4^+^ T cells. Taken together, these results lead us to speculate that this cell population might also expand during other infections. This could explain the variation observed in the pre-existing proportion of CD38^+^ cells among total CD4^+^ T cells between individuals (ranging from 0.9 to 10.9% of total CD4^+^ T cells), reflecting a more or less recent infection with a pathogen. This idea is also supported by the recent study from Tsang et al, which highlighted that the proportion of CD4^+^ T cells expressing CD38 was a feature presenting very high inter-individual variability [63].

In conclusion, we have identified a unique population of circulating CD38^+^ CD4^+^ T cells in humans with characteristics that clearly distinguish them from other CD4^+^ T cells; specifically, markers of recent activation and cytotoxic potential but impaired cytokine production. This CD4^+^ T cell population was specifically expanded in experimental blood-stage malaria infection and was significantly associated with reduced parasite burden. This is consistent with a critical role for these cells in protective immunity to malaria. Further insights into the function and origin of this cell population might provide novel immune correlates of protection in blood-stage malaria and other infectious diseases, and inform vaccine design.

## Material and Methods

### Ethics statement

Experimental infection of malaria-naive healthy adult volunteers was undertaken at QPharm Pty Ltd (Brisbane, Australia) with approval of the QIMR Berghofer Medical Research Institute Human Research Ethics Committee (QIMRB-HREC) and Medicines for Malaria Venture (MMV). Clinical studies were registered on the Australian and New Zealand Clinical Trials Registry (ANZCTR): clinical trial numbers ACTRN12612000814875, ACTRN12613000565741 and ACTRN12613001040752. All subjects were enrolled with written informed consent.

### Samples from *P*. *falciparum* experimentally infected volunteers

Inoculum preparation, volunteer recruitment, infection, monitoring and treatment were performed as described previously [64]. In brief, healthy malaria-naive individuals were intravenously inoculated with 1,800 viable *P*. *falciparum* parasitized erythrocytes, and treated with anti-malarial drugs at seven to eight days post-infection. Blood samples were collected prior to infection and seven days post-infection (before anti-malarial drug treatment). Peripheral blood from healthy volunteers was also collected under informed consent and approval by the QIMRB-HREC. Peripheral blood collected in Lithium Heparin Vacutainers (BD Biosciences) was either used directly for flow cytometry analysis, or peripheral blood mononuclear cells (PBMC) isolated using standard Ficoll density gradient centrifugation.

### Determination of parasite burden in *P*. *falciparum* experimentally infected volunteers

Parasitemia levels over the course of the infection were determined using a consensus *P*. *falciparum* species-specific quantitative PCR assay from 500 μl of packed red blood cells as previously described [65]. Parasite levels were assessed once daily until day four post-infection and then twice daily until treatment. All samples were batch tested in triplicate together after each study completion. The total parasite burden across the first seven days of infection was defined as the area under the curve of the transformed parasite levels from day 0 to day 7 of infection using the trapezoidal rule. The limit of detection was 64 parasites/500 μl packed red blood cells [65].

### Flow cytometry antibodies and staining buffer

Details about the clones, manufacturers and optimum dilutions for each antibody used in this study are listed in S1 Table. Unless otherwise stated, staining buffer was a PBS solution supplemented with 0.5% FCS and 4 mM EDTA and filtered through 0.2 μm.

### Flow cytometric analysis from whole blood

Whole blood collected in Lithium Heparin vacutainers was lysed and fixed with BD FACS lysing solution (BD Biosciences) and lymphocytes permeabilized with BD FACS permeabilizing solution 2 (BD Biosciences) according to the manufacturer’s instructions. Cells were then resuspended in 50 μl of staining buffer containing surface and intracellular antibodies at previously determined optimum dilution along with 1 μl of human Fc receptor blocking solution (Human TruStain FcX, Biolegend) for 30 mins at room temperature, washed and resuspended in staining buffer. Samples were acquired using a LSR Fortessa 4 (BD Biosciences) with Diva software, and analyzed using FlowJo software (version 6.0).

### Flow cytometric analysis from PBMC and sorted CD4^+^ T cells

Approximately 5x10^5^ cells were washed in PBS, resuspended in 100 μl PBS containing 0.1 μl Zombie Yellow fixable viability dye (Biolegend) and incubated for 15 mins at 4°C. Cells were then washed, resuspended in 20 μl of staining buffer containing surface antibodies at previously determined optimum dilution along with 1 μl of human Fc receptor blocking solution for 20 mins at 4°C, fixed in 50 μl of 1% paraformaldehyde solution for 15 mins at room temperature, washed, resuspended in 15 μl of BD Perm/Wash buffer 1x (BD Biosciences) containing intracellular antibodies at previously determined optimum dilution for 30 mins at 4°C, washed with BD Perm/Wash 1x solution, and resuspended in staining buffer. Samples were acquired using a LSR Fortessa 4 (BD Biosciences) with Diva software, and analyzed using FlowJo software (version 6.0).

### Phosphoflow on whole blood

200 μl of whole blood was stained with anti-human CD4-BV510, anti-human CD8-APC-H7 and anti-human CD38-APC at previously determined optimum dilution for 15 mins at 37°C with or without recombinant human IFN-α2 (Biolegend) or recombinant human IFN-γ (ProSpec) at 100 ng/mL. Whole blood was lysed and fixed with Phosflow Lyse/fix 1x solution (BD Biosciences) and lymphocytes permeabilized with BD Perm III solution (BD Biosciences) according to the manufacturer’s instructions. Cells were then resuspended in 50 μl of staining buffer containing anti-human pSTAT1-PECF594, anti-human pSTAT4-AF488 and anti-human pSTAT5-PE at previously determined optimum dilution for one hour at room temperature. Cells were washed and resuspended in staining buffer. Samples were acquired using a LSR Fortessa 4 (BD Biosciences) with Diva software, and analyzed using FlowJo software (version 6.0).

### Cell sorting

CD38^+^ and CD38^-^ CD4^+^ T cells were sorted from freshly isolated or cryopreserved PBMC. Approximately 20x10^6^ PBMC were resuspended in 50 μl of staining buffer containing anti-human CD4-BV510, anti-human CD8-APC-H7 and anti-human CD38-PerCpCy5.5 at previously determined optimum dilution for 20 mins at 4°C, washed and resuspended in staining buffer. Just before the sorting, 1 μg/mL of propidium iodide (Sigma-Aldrich) was added to allow for assessment of viability. Pi^-^CD4^+^CD8^-^CD38^+^ and Pi^-^CD4^+^CD8^-^CD38^-^ cells were sorted using a BD Aria III cell sorter (BD Biosciences) directly in staining buffer and kept on ice until further use for *in vitro* assays.

### Gene expression on *ex vivo* or *in vitro* stimulated sorted cells

RNA was extracted from sorted CD38^+^ and CD38^-^ CD4^+^ T cells directly *ex vivo* by resuspending the cells directly in RLT buffer or after *in vitro* stimulation to induce cytokine production. For the *in vitro* stimulation assay, sorted cells were plated at 50,000 cells/well in RPMI 1640 containing 25 mM Hepes, 2 mM L-glutamine (Invitrogen), and supplemented with 10 units/mL of Penicillin (Life Technologies), 10 μg/mL of Streptomycin (Life Technologies) and 10% fetal bovine serum (Life Technologies) and stimulated with 5 ng/mL of phorbol myristate acetate PMA (Sigma Aldrich) and 500 ng/mL Ionomycin (Sigma Aldrich) for 5h at 37°C in an atmosphere of 5% C0_2_. Following stimulation, supernatants were harvested, cell pellets resuspended in RLT buffer and stored at -70°C. On the day of extraction, frozen cell lysates were thawed quickly on ice and mRNA was extracted using the RNEasy micro kit (Qiagen) according to the manufacturer’s instructions. cDNA was synthesized using oligo-dT and Superscript III RT (Invitrogen) according to the manufacturer’s instructions. Eomes, Granzyme B, Granzyme A, Granzyme K, IFN-γ, IL-2, IL-4, IL-10, IL-17A, Perforin, RPL13A, and TNF gene expression were measured from 1 μl of cDNA (or pre-amplified cDNA) using individual Taqman gene expression assays (Life Technologies) and Platinum Taq polymerase (Life technologies) in a 10 μl volume reaction on a 384-well plate using the Light Cycler 480 real time PCR cycler (Roche) according to the manufacturer’s instructions. Each gene expression was assayed in duplicate. Cycling conditions used were: 50°C for 2 mins followed by 95°C for 10 mins, 40 cycles at 95°C for 15 secs and 60°C for 1 min. Fold changes were calculated with the ddCt value method [66] using RPL13A expression as the reference gene, and the expression values from the unstimulated cells as baseline.

### Intracellular cytokine staining on sorted cells

Sorted CD38^+^ and CD38^-^ CD4^+^ T cells were plated at 50,000 cells/well in RPMI 1640 containing 25 mM Hepes, 2 mM L-glutamine, supplemented with 10 units/mL of Penicillin, 10 μg/mL of Streptomycin and 10% fetal bovine serum and stimulated with 5 ng/mL of phorbol myristate acetate and 500 ng/mL Ionomycin for 5h at 37°C in an atmosphere of 5% C0_2_. After one hour, Brefeldin A (GolgiPlug, BD Biosciences) was added at 1 μg/ml. Following stimulation, cells were washed, resuspended in 20 μl of staining buffer containing anti-human CD4-BV510 and anti-human CD8-APC-H7 at previously determined optimum dilution for 20 mins at 4°C, fixed in 50 μl of 1% paraformaldehyde solution for 15 mins at room temperature, washed, resuspended in 15 μl of BD Perm/Wash buffer 1x (BD Biosciences) containing anti-human IFN-γ-FITC at previously determined optimum dilution for 30 mins at 4°C, washed with BD Perm/Wash 1x solution, and resuspended in staining buffer before acquisition on LSR Fortessa 4 (BD Biosciences) with Diva software. FlowJo version 6.0 was used for gating.

### TCR stimulation assay on sorted cells

Sorted CD38^+^ and CD38^-^ CD4^+^ T cells were plated at 50,000 cells/well in RPMI 1640 containing 25 mM Hepes, 2 mM L-glutamine, and supplemented with 10 units/mL of Penicillin, 10 μg/mL of Streptomycin and 10% fetal bovine serum in a 96-well plate pre-coated overnight with 10 μg/mL of anti-human CD3 OKT3 antibody (Biolegend) and incubated for 5 h at 37°C in an atmosphere of 5% C0_2_. Following stimulation, cells were resuspended in 20 μl of staining buffer containing anti-human CD4-BV510, anti-human CD8-APC-H7, anti-human CD69-AF700 at previously determined optimum dilution for 20 mins at 4°C, washed and resuspended in staining buffer before acquisition on LSR Fortessa 4 (BD Biosciences) with Diva software. FlowJo version 6.0 was used for gating.

### *In vitro* generation of CD38^+^ CD4^+^ T cells

Sorted CD38^-^ CD4^+^ T cells from healthy uninfected volunteers were plated at 0.5x10^6^ cells/well in RPMI 1640 containing 25 mM Hepes, 2 mM L-glutamine, supplemented with 10 units/mL of Penicillin, 10 μg/mL of Streptomycin and 5% human AB serum (Sigma Aldrich) and stimulated with *P*. *falciparum* parasitized red blood cells extract at 1x10^6^ pRBC/ml or PHA at 5 μg/mL (Sigma Aldrich) for six days at 37°C in an atmosphere of 5% C0_2_. Following stimulation, cells were either used directly for flow cytometric analysis, or further sorted into CD38^+^ and CD38^-^ cells for *in vitro* stimulation and gene expression analysis as described above.

### Statistical analysis

Statistical analyses were performed using GraphPad Prism Software (version 6). Normality was assessed using D’Agostino and Pearson Omnibus normality test and showed the datasets were not normally distributed. Therefore, all paired datasets were compared using the non-parametric Wilcoxon test. All univariate and multivariate analysis were performed with R. Univariate analysis was performed with a linear regression analysis and *p* values were adjusted for multiple comparisons using the Bonferroni method. Multivariate analysis was performed with a multivariate linear regression analysis with forward stepwise covariate selection using Akaike information criteria. *P* values less than 0.05 were considered as significant.

## Supporting Information

S1 Table Antibodies used for flow cytometry. (DOCX)

S1 Fig Absolute CD69+ and CD38+ T cell counts during P. falciparum experimental blood-stage infection. Peripheral blood was collected prior to and seven days post-infection. Absolute lymphocyte counts were determined by full blood count and the frequency of CD69^+^ or CD38^+^ T cells and B cells determined by flow cytometry. Graph show combined data from 22 volunteers from five independent cohorts; statistical differences between pre- and post-infection were determined using the non-parametric Wilcoxon test; box and whisker plots indicate median, interquartile range and min-max; ns, *p* > 0.05.
(TIF)

S2 Fig Absolute CD4^+^ T cell counts during *P*. *falciparum* experimental blood-stage infection. Peripheral blood was collected prior to and seven days post-infection. Absolute lymphocyte counts were determined by full blood count and the frequency of CD4^+^ T cells amongst lymphocytes determined by flow cytometry. Graph show combined data from 22 volunteers from five independent cohorts; statistical differences between pre- and post-infection were determined using the non-parametric Wilcoxon test; box and whisker plots indicate median, interquartile range and min-max; ns, *p* > 0.05.
(TIF)

S3 Fig Cytokine production by *in vitro* generated CD38^+^ CD4^+^ T cells from CD38^-^ CD4^+^ T cells. CD38^+^ CD4^+^ T cells were generated *in vitro* from CD38^-^ CD4^+^ T cells isolated from peripheral blood of healthy volunteers by *in vitro* stimulation with *P*. *falciparum* parasitized red blood cells extract (pRBC) at 10^6^ pRBC/mL for 6 days. Their cytokine gene expression was measured by RT-qPCR after brief mitogenic stimulation with PMA and Ionomycin. Gene expression was normalized to reference gene RPL13A. Graphs show combined data from four volunteers. Box and whisker plots indicate median, interquartile range and min-max.
(TIF)
