# Supplementary material for: Reduced Plasmodium Parasite Burden Associates with CD38+ CD4+ T Cells Displaying Cytolytic Potential and Impaired IFN-γ Production
Source: PLoS Pathog. 2016 Sep 23;12(9):e1005839. doi: 10.1371/journal.ppat.1005839 (PMC5035011; doi:10.1371/journal.ppat.1005839)
Supplement: S1 Table — (DOCX) [file ppat.1005839.s001.docx]

**S1 Table. Antibodies used for flow cytometry**

| **Category** | **Antigen** | **Fluorochrome** | **Clone** | **Manufacturer** | **Dilution** |
| --- | --- | --- | --- | --- | --- |
| Surface | CD3 | PE | HIT3a | Biolegend | 1/200 |
|  | CD4 | AF488 | RPA-T4 | BD Biosciences | 1/100 |
|  | CD4 | BV510 | OKT4 | Biolegend | 1/200 |
|  | CD8 | APC-H7 | SK1 | BD Biosciences | 1/400 |
|  | CD14 | AF594 | HCD14 | Biolegend | 1/400 |
|  | CD19 | PE-Cy7 | HIB19 | Biolegend | 1/200 |
|  | CD25 | AF700 | BC96 | Biolegend | 1/50 |
|  | CD38 | APC | HIT2 | Biolegend | 1/400 |
|  | CD38 | PerCpCy5.5 | HB-7 | Biolegend | 1/400 |
|  | CD56 | PE-Cy7 | B159 | Biolegend | 1/50 |
|  | CD69 | AF700 | FN50 | Biolegend | 1/100 |
|  | Nkp46 | BV421 | 900 | Biolegend | 1/200 |
|  | TCRαβ | PE-Cy5 | IP26 | Biolegend | 1/400 |
|  | TCRγδ | APC | B1 | Biolegend | 1/50 |
|  | TCRVα24Jα18 | AF488 | 6B11 | Biolegend | 1/100 |
| Intracellular | Ki67 | PerCp Cy5.5 | B56 | Biolegend | 1/400 |
|  | Granzyme B | Pac Blue | GB11 | Biolegend | 1/400 |
|  | Perforin | PE | dG9 | Biolegend | 1/400 |
|  | IFN-γ | FITC | B27 | BD Biosciences | 1/100 |
|  | Bcl2 | AF647 | 100 | Biolegend | 1/30 |
|  | STAT1 | PE | 1/Stat1 | BD Biosciences | 1/20 |
| Phosphorylated | pSTAT1 (pY701) | PECF594 | 4a | BD Biosciences | 1/20 |
|  | pSTAT4 (pY693) | AF488 | 38/p-Stat4 | BD Biosciences | 1/10 |
|  | pSTAT5 (pY694) | PE | 47 | BD Biosciences | 1/10 |
