# Supplementary figures and images for: Reduced Plasmodium Parasite Burden Associates with CD38+ CD4+ T Cells Displaying Cytolytic Potential and Impaired IFN-γ Production
Source: PLoS Pathog. 2016 Sep 23;12(9):e1005839. doi: 10.1371/journal.ppat.1005839 (PMC5035011; doi:10.1371/journal.ppat.1005839)

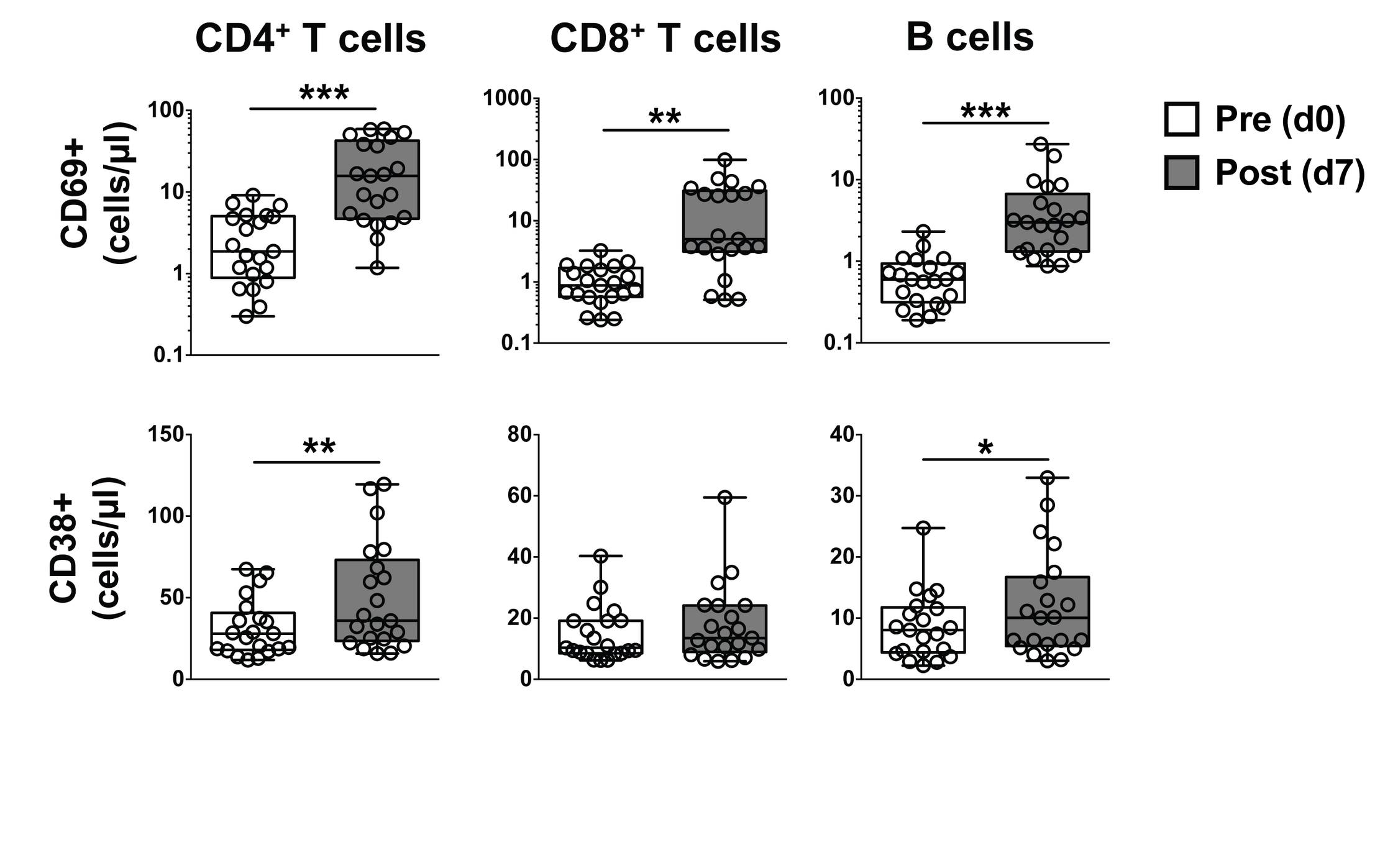

Supplement: S1 Fig — Peripheral blood was collected prior to and seven days post-infection. Absolute lymphocyte counts were determined by full blood count and the frequency of CD69+ or CD38+ T cells and B cells determined by flow cytometry. Graph show combined data from 22 volunteers from five independent cohorts; statistical differences between pre- and post-infection were determined using the non-parametric Wilcoxon test; box and whisker plots indicate median, interquartile range and min-max; ns, p > 0.05. (TIF) [file ppat.1005839.s002.tif]

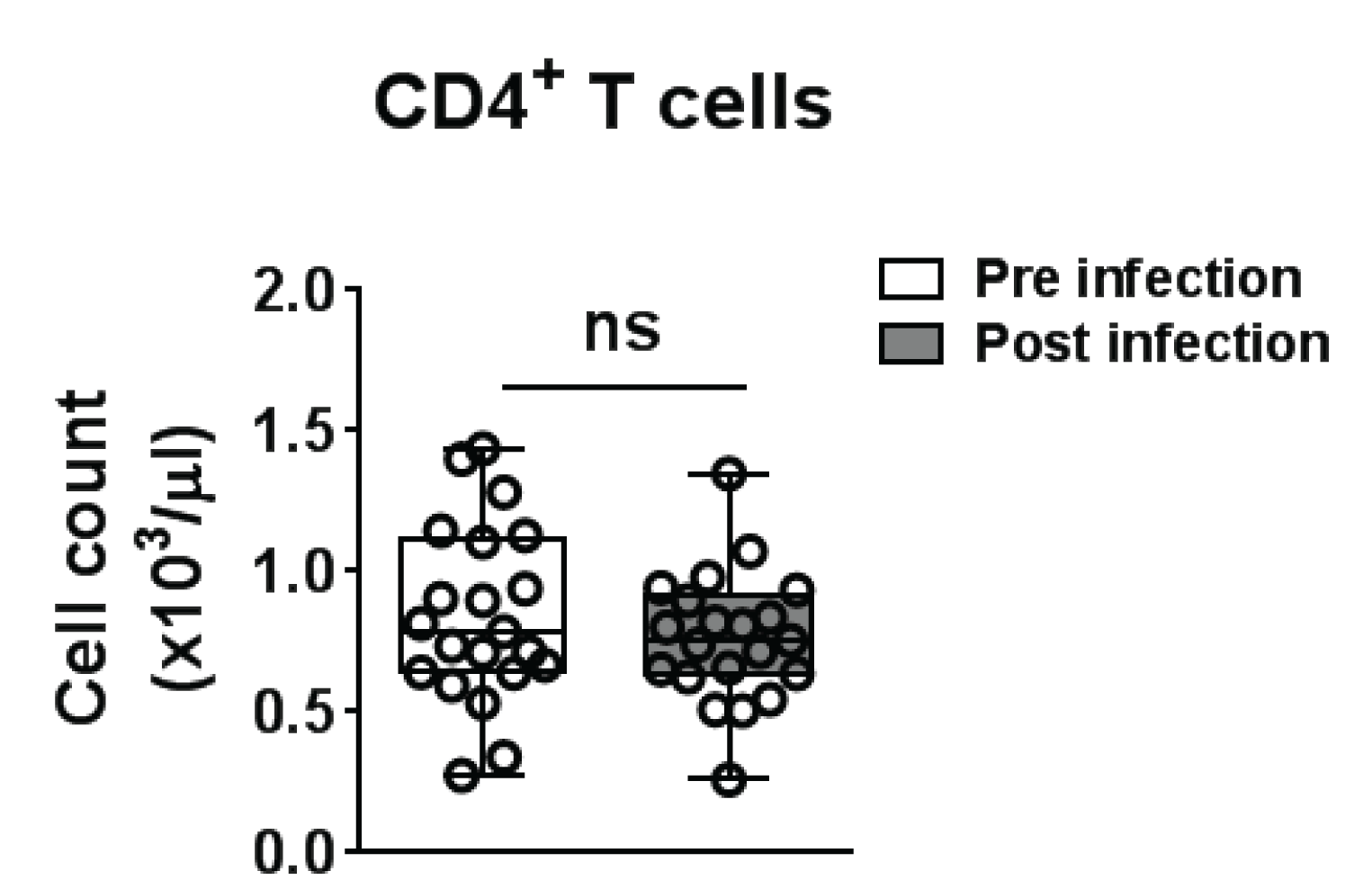

Supplement: S2 Fig — Peripheral blood was collected prior to and seven days post-infection. Absolute lymphocyte counts were determined by full blood count and the frequency of CD4+ T cells amongst lymphocytes determined by flow cytometry. Graph show combined data from 22 volunteers from five independent cohorts; statistical differences between pre- and post-infection were determined using the non-parametric Wilcoxon test; box and whisker plots indicate median, interquartile range and min-max; ns, p > 0.05. (TIF) [file ppat.1005839.s003.tif]

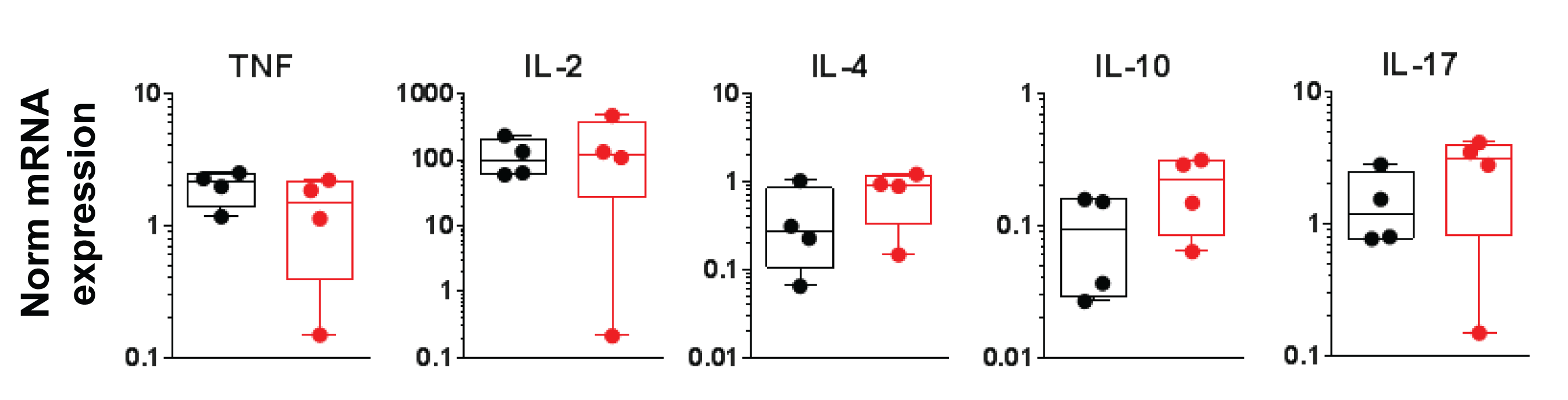

Supplement: S3 Fig — CD38+ CD4+ T cells were generated in vitro from CD38- CD4+ T cells isolated from peripheral blood of healthy volunteers by in vitro stimulation with P. falciparum parasitized red blood cells extract (pRBC) at 106 pRBC/mL for 6 days. Their cytokine gene expression was measured by RT-qPCR after brief mitogenic stimulation with PMA and Ionomycin. Gene expression was normalized to reference gene RPL13A. Graphs show combined data from four volunteers. Box and whisker plots indicate median, interquartile range and min-max. (TIF) [file ppat.1005839.s004.tif]
